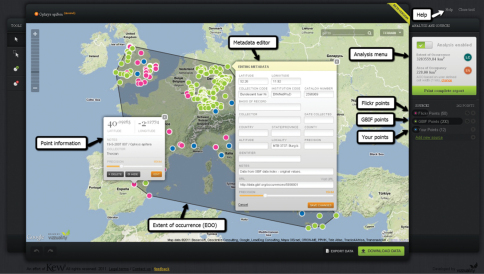# Supporting Red List threat assessments with GeoCAT: geospatial conservation assessment tool

**DOI:** 10.3897/zookeys.150.2109

**Published:** 2011-11-28

**Authors:** Steven Bachman, Justin Moat, Andrew W. Hill, Javier de la Torre, Ben Scott

**Affiliations:** 1 Royal Botanic Gardens, Kew, UK; 2 Vizzuality, Spain; 3 Natural History Museum, UK

**Keywords:** Red List, Conservation, Open Source, Biodiversity, Mapping, IUCN, GBIF, Flickr, Geospatial, Google maps, HTML5, JSON, AJAX

## Abstract

GeoCAT is an open source, browser based tool that performs rapid geospatial analysis to ease the process of Red Listing taxa. Developed to utilise spatially referenced primary occurrence data, the analysis focuses on two aspects of the geographic range of a taxon: the extent of occurrence (EOO) and the area of occupancy (AOO). These metrics form part of the IUCN Red List categories and criteria and have often proved challenging to obtain in an accurate, consistent and repeatable way. Within a familiar Google Maps environment, GeoCAT users can quickly and easily combine data from multiple sources such as GBIF, Flickr and Scratchpads as well as user generated occurrence data. Analysis is done with the click of a button and is visualised instantly, providing an indication of the Red List threat rating, subject to meeting the full requirements of the criteria. Outputs including the results, data and parameters used for analysis are stored in a GeoCAT file that can be easily reloaded or shared with collaborators. GeoCAT is a first step toward automating the data handling process of Red List assessing and provides a valuable hub from which further developments and enhancements can be spawned.

## Introduction

Recent estimates suggest there could be 8.7 million (± 1.3 million) species on the planet (Mora et al. 2011). Even at the lowest estimate, less than 1% (61,914, IUCN 2011) of those species have been formally assessed using the Red List system to determine their conservation status i.e. an assessment of the risk that they will become extinct. A key factor in the lack of progress in the production of species conservation assessments is the scarcity of user friendly, but powerful, analytical tools which are readily available to scientists and communities to carry out these assessments. Furthermore, large amounts of primary biodiversity data are now available via services such as the Global Biodiversity Information Facility (GBIF), but have yet to be fully utilised for conservation action. With the trend in biodiversity loss increasing across the globe (Secretariat of the Convention on Biological Diversity 2010) it is essential that we speed up the production of assessments. This will enable us to more quickly identify species and regions at greatest risk so that it may guide conservation action. To scale up the production of conservation assessments to the level of mega-diverse groups such as plants and insects, there needs to be significant progress in the development of automated and semi-automated techniques that scientists and other experts can harness. Here, we present the Geospatial Conservation Assessment Tool (GeoCAT - http://geocat.kew.org), which is a first step towards that goal.

## Methods

### Analysis using GeoCAT

**Figure1.**
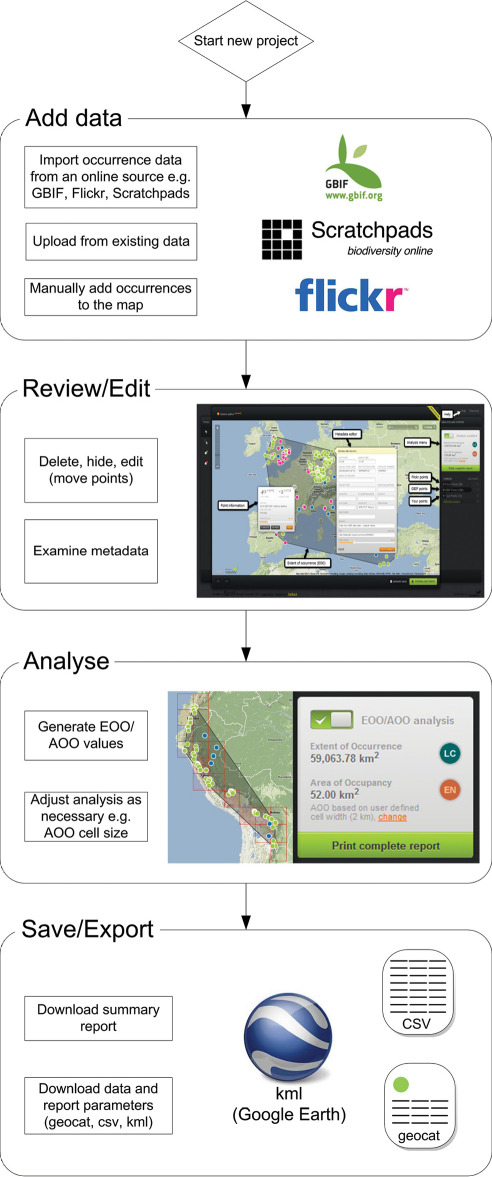
GeoCAT workflow; Start a new project and add data to the map via the three options. Existing data may be derived from an output of an existing database or from an online source such as GBIF, Flickr or Scratchpads. Alternatively, click directly on the map to create markers to signify the occurrence of the taxon you wish to assess. The intuitive mapping interface allows interaction with the data to delete, move or hide points from analysis. The metadata window exposes the attributes of the occurrences e.g. date of collection, collector, location and provides a direct link to the raw data.<br/> After editing the data the analysis can be enabled and the results are displayed as grpahics on the map and through a report window. The EOO/AOO values, preliminary IUCN categories and parameters are shown. AOO cell size can be adjusted. Statistics generated from the analysis and a basic map can be downloaded as a report. Occurrence data used in the analysis can be downloaded as a kml file for integration with Google Earth or as a CSV file. In addition, a single geocat. file encompassing all analysis results, parameters, map settings and occurrence data can be saved for later use, or to pass to collaborators for additional work.

GeoCAT can be accessed from the following URL: http://geocat.kew.org/. The tool was developed to utilise spatially referenced primary occurrence data to analyse two aspects of the geographic range of a taxon: the extent of occurrence (EOO) and the area of occupancy (AOO). These two measures are the foundation of the ‘B’ criterion of the IUCN Red List system (IUCN 2001) - see ‘*Technology and algorithms*’ section below for full definition of EOO and AOO. Figure 1 illustrates how GeoCAT users can quickly and easily add, review, edit and analyse data and finally save and export the results.

### Technology and algorithms

GeoCAT is built using the latest web-technologies based in JavaScript and HTML5. The result is a responsive and intuitive environment for web-based GIS and conservation analysis algorithms. The tool was built to combine private data provided by the user, public resources such as Flickr, and scientific resources such as GBIF. GeoCAT makes importing geospatial species data simple, by either searching and loading data from the online sources or importing and mapping CSV files. The Google Maps API and the custom user interface provide a high quality map environment to perform geographic analysis of data location and its quality; the user can delete or move data individually or through filters (e.g. drawing bounding boxes) also defining thresholds for common components of the data such as coordinate precision. Algorithms for measuring species threat are implemented directly in the browser, avoiding any need to move data to desktop applications or to send the data for server-side processing. The GeoCAT file format streamlines the process of restarting a project by encoding all data, including algorithm parameters, outputs, and application state, into a web syntax called JSON. The file can then be stored by a user for sharing or later use.

The inclusion of external data from GBIF and Flickr was an important feature for bringing a robust species assessment tool to the web. To achieve this functionality, GeoCAT relies on cross domain AJAX requests, where the application in the user’s browser directly queries, receives, and parses data from the external sources. Therefore the application relies heavily on consistent data standards, where the data received will be in a predictable format. For example, GBIF provides a REST API where data can be queried and downloaded, the data standards are encoded in their web-services documentation, http://data.gbif.org/ws/. Georeferenced images from Flickr are queried using machine tags and a keyword search.

GeoCAT presently uses two algorithms to calculate EOO and the AOO (after Willis et al. 2003). These were originally developed in the Avenue scripting language for ArcView 3.3 within the Conservation Assessment Tools (CAT) extension (developed at the Royal Botanic Gardens, Kew and downloadable from: http:// www.kew.org/gis/projects/cats (Moat 2007)), these were reprogrammed in JavaScript for GeoCAT.

**Figure 2.**
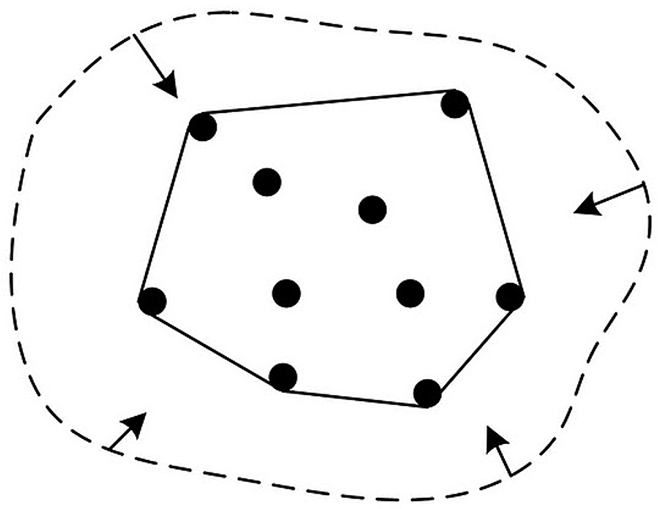
Illustration of a convex hull of a set of points. Imagine stretching a rubber band so that all points are inside it, then releasing it; when it becomes tight, the area enclosed is the convex hull.

EOO is a measure of the geographic range size of a species. One of the simplest methods to calculate this is a convex hull which is defined as the smallest polygon in which no internal angle exceeds 180˚ and contains all sites of occurrence (see Figure 2). There are many algorthims developed to calculate the convex hull from a set of points, but within GeoCAT we use a quickhull (Eddy 1977 and Bykat 1978) with code developed from Echo 2 (http://blogs.infoecho.net/echo/2007/03/) and Eriestuff (http://eriestuff.blogspot.com/2008/03/google-maps-convex-hull-of-point-set-or.html)

AOO is a measure of the area in which a species occurs. One of the more straightforward ways of measuring this is to sum the area of square grids the species occupies. There is much discussion on the influence of scale and what cell size is appropriate (Kunin and Hartley 2003, Willis et al. 2003, Callmander et al. 2007). IUCN states that: ‘‘the most appropriate scale will depend on the taxon in question, and the origin and comprehensiveness of the distribution data’’ (IUCN Standards and Petitions Subcommittee 2010). Within GeoCAT we have allowed the user to choose the cell size using three methods. The default is 2km^2^ cell size (as recommended in the IUCN guidelines - IUCN 2010), user defined cell size and finally 1/10th of the maximum distance between the most distance pair of points (Willis et al. 2003). The last method uses a factor of 10 as this reflects the relationship between EOO and AOO in the IUCN criteria and gives a size of the grid reflecting the geographic scale of the species distribution. Cells are calculated using simple maths to degrade each point to the lower left corner of the cell ((Floor ((x or y)/cellwidth) * cellwidth )), cells are then constructed from this lower left corner. In addition, the number of points within the cell are recorded and used to colour the cell on the map to give an indication of density of collections.

### Open source

GeoCAT was developed as an open source tool. This means that the methods and contributions of the code itself can help inform the informatics community in the future. Open source also aids in the transparency of decision making, by allowing anyone to see and audit algorithms. The code is accessible to anyone from the project’s Github repository (https://github.com/Vizzuality/GeoCAT). We hope that this will help lower the cost of attracting new algorithms and community developed solutions with the tool.

### Scratchpad integration

GeoCAT relies on primary occurrence data to drive the analysis. One of the major new platforms for primary biodiversity data is the Scratchpads project (Smith et al. 2009). With 281 sites across a broad spectrum of natural history science (including the lesser known groups where their conservation status is poorly known) and thousands of primary data records, the Scratchpads project is an obvious choice for integration with GeoCAT. Scratchpad users will be able to access specimen or occurrence data directly from GeoCAT. Similar to the GBIF and Flickr ‘source data’ options it is possible to query data from a specific Scratchpad site to directly access and plot specimen data for analysis. In addition, from within a Scratchpad site, users will be able to open GeoCAT directly from a Scratchpad page where structured specimen or occurrence data exists. This will instantly display the data, assuming it contains georeferenced records. Finally, users will be able to upload a .geocat report file to a Scratchpad page. From here a URL link can take the user back to GeoCAT site where further analysis can be performed. In summary:

• GeoCAT will be able to access and import Scratchpad specimens via a new web service to output structured data in Darwin Core format.

• GeoCAT can be opened directly from within a scratchpad page, using a link encoded with URL parameters to retrieve the structured data source.

• Users should be able to upload a .geocat file report in a scratchpad page and the page will offer the option to open it in GeoCAT.

Other systems containing large amounts of primary data such as BRAHMS (http://dps.plants.ox.ac.uk/bol/BRAHMS/Home/Index) have also integrated with GeoCAT by supporting the export of a compatible CSV file.

### Caveats

It is intended that the tool is utilised primarily by those wishing to carry out Red List conservation assessments, although it also functions well as a simple web mapping tool for other uses such as georeference checking. It is expected that the user has a good understanding of the taxa being assessed, the quality of the underlying data and a good knowledge of the Red List criteria. GeoCAT can provide metrics that partially fulfil criterion B assessments and allow a preliminary rating to be obtained. In order to complete a full Red List assessment a number of additional sub-criteria must be met and a minimum set of data are required to accompany the assessment. For further information see the IUCN Red List technical documents: (http://www.iucnredlist.org/technical-documents/data-organization).

It is not within the scope of this paper to discuss the use of EOO and AOO for Red List assessments as this has been considered elsewhere (see IUCN Standards and Petitions Subcommitte 2010 and references therein). It is hoped that complementary algorithms such as Alpha hulls (α-hulls - generalisations of convex hulls) can be incorporated into later versions of GeoCAT to provide the user with a wider range of options for a more robust analysis. The use of α-hulls may be a more appropriate method for investigating reductions or continuing declines in EOO (IUCN 2010).

At present there are some limitations on number of occurrence records that can be displayed from both GBIF (500) and Flickr (250). Users will be informed if their query returns more points than the display limit. Initial performance tests suggest the map display can handle many thousands of points, but further testing is needed. The records displayed are the first to be queried, but it is hoped that later versions of GeoCAT will provide further refinements to this query e.g. most recently collected records first or those with highest georeference precision.

## Conclusion

### Future directions

It is anticipated that significant improvements will be made for later versions of GeoCAT. An obvious shortcoming is that the tool only deals with one aspect of the IUCN criteria and can only report a preliminary assessment based on the two range measures extent of occurrence (EOO) and area of occupancy (AOO). An obvious extension is to incorporate additional range based analysis that can inform other aspects of the IUCN criteria such as number of locations, sub-populations and degree of fragmentation.

Although the tool is geospatial in its focus, there is the opportunity to extend analysis into the temporal elements of museum specimen data, such as the date of collection. Statistical approaches have already been investigated (Solow and Roberts 2003, McPherson and Myer 2009, Collen et al. 2010) and could simply be modified for inclusion in GeoCAT as an additional module. Examining occurrence data through time could open up other parts of the Red List criteria such as Criterion A that deals with ‘reduction’ or decline in population size. For example within GeoCAT historical specimens can be removed when they occur in areas known to have been subject to recent habitat loss. Reductions in EOO and AOO can then be recorded and potentially applied to Criterion A.

At present assessments can only be carried out one at a time. In order to scale up the production of assessments a batch option is needed whereby a single file of occurrence data for multiple species can be uploaded and processed. This would allow hundreds of assessments to be processed in a matter of seconds.

Further enhancements can also be made with regard to the handling of point data through the GBIF portal. The added bonus of the slick mapping interface makes GeoCAT a useful tool for georeference checking and cleaning. Querying the raw data from GBIF can often reveal obvious georeferencing mistakes such as outliers or swapped latitude and longitude pairs. The easy click and drag editing of points means they can be accurately placed on the map to ensure the most precise analysis. GeoCAT allows you to track which points have been edited, but at present there is no easy mechanism for feeding back this information to the original data provider – this could be a service integrated into the GBIF portal. Until this feedback loop is established the erroneously georeferenced records from data providers will continue to be served up by GBIF.

Harvesting of GBIF data also provides an opportunity to put the occurrence data of your target species in the context of the background collecting rate in a region. Presence of your target species i.e. the one you wish to assess is easy to determine with a verified record, but absence is more difficult. GBIF data can be used to determine a background collecting rate for your target group e.g. plants. Absence of your target species in an area with a high intensity of background sampling provides evidence that your target species may be absent.

An exciting potential extension of GeoCAT is to provide better integration with cloud based data such as Google Fusion tables. This could work in three ways: i) linking GeoCAT to specimen data stored in the cloud thereby allowing on-the-fly editing ii) exporting assessment results to tables in the cloud and iii) linking to custom layer data in kml/kmz format. This could lead to the first entirely automated cloud based conservation assessments.

The functionality of adding user generated kml/kmz files also offers significant potential. Threat datasets from fires to land cover change and deforestation can be added. At present the layer files can be visualised, but it is not possible to interact with the layers via spatial queries in the same was as a GIS. Adding this kind of functionality would instantly allow more rigorous data driven assessments.

### Benefits

GeoCAT provides a mechanism for data driven conservation assessments in a transparent, repeatable and rapid way through a user friendly environment. The benefits can be summarised as the following:

• Data driven assessments, giving an auditable data trail i.e. complete transparency of data used for assessments

• A simple, modern and easy to use interface.

• Accessible - opening up to assessors across the world - only an Internet connection is needed.

• Standardised, automated and repeatable analysis.

• Single-click analysis of Extent of Occurrence (EOO) and Area of Occupancy (AOO)

• Ability to import occurrence data from online sources such as GBIF or Flickr and other systems such as Brahms and Scratchpads. GeoCAT also allows export and reporting to other formats for further analysis or storage.

• Quick to use and easy to distribute data which can only accelerate the production of Red List assessments.

• Code is open source and development of algorithms are encouraged so the tool can develop towards a powerful automated assessment tool and for other geographic analysis.

GeoCAT responds directly to the growing need for more data driven analytical tools to aid the process of assessing species against the Red List criteria. The tool is intended to be a platform from which enhancements can be made and we encourage the developer community to engage with the GeoCAT project. We believe there are many exciting possibilities for the future development of GeoCAT. We hope GeoCAT can be utilised for the assessment of taxa at any spatial scale and across any taxonomic group, but especially those that are poorly represented on the Red List at present.

If you wish to acknowledge use of GeoCAT please use the following citation:

Bachman S, Moat J, Hill AW, de la Torre J, Scott B (2011) Supporting Red List threat assessments with GeoCAT: geospatial conservation assessment tool. In: Smith V, Penev L (Eds) e-Infrastructures for data publishing in biodiversity science. ZooKeys 150: 117–126. (version XX).

**Figure:**